# Multimodal MRI Analysis of Brain Metrics Associated with Depression Remission

**DOI:** 10.1192/j.eurpsy.2025.672

**Published:** 2025-08-26

**Authors:** G. Clain, M. Malestroit, J. Deverdun, E. Le Bars, V. Brand-Arpon, P. Courtet, D. Ducasse, E. Olie

**Affiliations:** 1 Emergency Psychiatry and Acute Care, Lapeyronie Hospital, CHU Montpellier; 2 Departement of Neuroradiology, Institut d’Imagerie Fonctionnelle Humaine I2FH platform, Gui de Chauliac, CHU Montpellier; 3 CNRS, INSERM, IGF, Montpellier University, Montpellier, France

## Abstract

**Introduction:**

Treatment-resistant depression is particularly challenging in individuals with a history of suicide, often associated with lower rates of remission. The antidepressant efficacy of Acceptance and Commitment Therapy (ACT) in individuals with a history of suicide has been demonstrated (Zhao B, et al. Ann Gen Psychiatry 2023;22:34). It is crucial to further understand the underlying mechanisms driving this therapeutic effect.

**Objectives:**

To investigate cerebral biomarkers of therapeutic response to Acceptance and Commitment Therapy (ACT) versus Progressive Relaxation Therapy (PRT) in depression and suicidal risk.

**Methods:**

This randomized controlled trial included 32 patients with a history of suicide attempts in the past year who underwent seven weekly sessions of ACT or PRT. They completed clinical and MRI examinations (resting state, diffusion tensor, cerebral blood flow measured by pCASL, and anatomical sessions) at baseline and after therapy completion. Resting-state functional changes (graph theory, functional connectivity) in two networks identified in previous depression studies (Zhang X, Xu R, Ma H, et al. Biol Psychiatry 2024;95:1091-1099), according to the Schaefer atlas, were measured between groups and in the entire sample. A Principal Component Analysis (PCA) was conducted to combine depression, hopelessness, and psychological pain into a single composite variable, explaining 77.8% of the variance with strong correlations to the original variables: r = .91. This Depression Component (DC) was used in further analyses.

**Results:**

The patients were predominantly women (87.5%) with a mean age of 40 years (SD = 12). 81% of patients experienced current depression. No significant Group x Time interaction was found. In the whole sample, no anatomy nor diffusion tensor metrics modification has been found. However, the reduction of the DC was negatively correlated with modularity (r = -0.476, pFDR < 0.05) for the Salience Ventral Attentional. Moreover, within this network the DC variable was negatively correlated with the cerebral blood flow of one region (r = -0.5, pFDR < 0.05) and positively correlated with functional connectivity (T = 4.72, pFDR < 0.05) (Figure 1).

**Image 1:**

**Figure:**
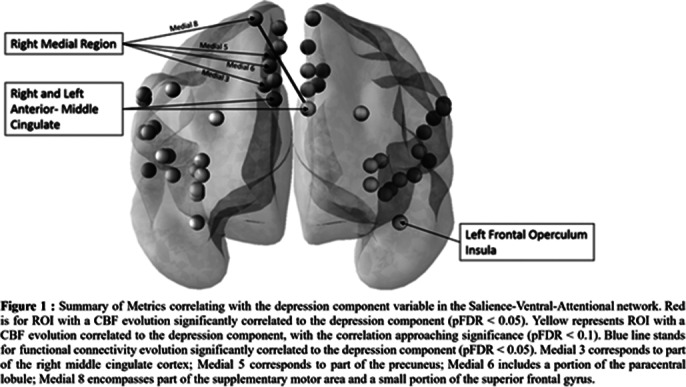

**Conclusions:**

These results suggest that the therapeutic response, and consequently the reduction of the depression component, might be related to a reorganization within the salience network. Modularity refers to how well a brain network is organized into distinct groups or clusters, where connections within each group are stronger than those between different groups. We observed increased modularity and cerebral blood flow within the salience ventral attentional network, which could reflect a more specialized and segregated functional organization. This reorganization could enhance the brain’s ability to regulate emotional and cognitive processes, supporting recovery from depression.

**Disclosure of Interest:**

None Declared

